# Association between bone turnover markers, bone mineral density, and serum osteoglycine in middle-aged men with Type 2 Diabetes mellitus

**DOI:** 10.1186/s13098-024-01388-8

**Published:** 2024-07-09

**Authors:** Salma Mohamed Mostafa, Ibrahim Elebrashy, Hemmat El Haddad, Olfat Shaker, Naglaa Abdel Razek, Ahmed Fayed

**Affiliations:** 1 Endocrinology Unit, Internal Medicine Department, Kasr Alainy School of Medicine, Cairo University, Giza, Egypt; 2 Medical Biochemistry and Molecular Biology Department, Kasr Alainy School of Medicine, Cairo University, Giza, Egypt; 3 Diagnostic and Interventional Radiology Department, Kasr Alainy School of Medicine, Cairo University, Giza, Egypt; 4 Nephrology Unit, Internal Medicine Department, Kasr Alainy School of Medicine, Cairo University, Giza, Egypt

**Keywords:** Diabetes mellitus, Bone mineral density, Bone turnover markers, DEXA, Serum osteoglycin

## Abstract

**Background:**

Patients with Type 2 diabetes mellitus (T2DM) have decreased bone health. We aimed to investigate serum levels of bone turnover markers (BTMs) (markers of bone formation and bone resorption) and bone mineral density (BMD) at three sites (lumber, neck femur, and total femur) in middle-aged men with type 2 diabetes and to analyze the relationship between them. Also to evaluate serum osteoglycin as a novel marker and its relation to BTMs, BMD, and diabetic status.

**Methods:**

We recruited seventy-eight patients with T2DM and thirteen non-diabetic, male volunteers as a control group. BMD was measured using a DEXA scan. BTMs (carboxy-terminal crosslinking telopeptide of type 1 collagen [CTX] and procollagen type 1 N propeptide [P1NP]), osteoglycin, PTH, and vitamin D were estimated. Data was compared among subjects and statistical analysis was performed.

**Results:**

Most of the patients were having normal BMD with no significant difference between patients and the controls. BTMs and osteoglycin were significantly higher and vitamin D was significantly lower in the diabetic patients. Serum osteoglycin was positively correlated with DEXA Neck Femur (*r* = 0.233; p-value < 0.05).

**Conclusion:**

Body mass index and Serum osteoglycin have a significant positive effect on BMD. Both markers of bone formation and bone resorption were increased indicating a state of increased bone turnover in T2DM.

## Introduction

Diabetes is a complex, chronic illness requiring continuous medical care with multifactorial risk reduction strategies beyond glycemic control. Patients with type 2 diabetes mellitus (T2DM) are at risk for multiple complications, such as macro- and micro-vascular disease. Recently, an increased risk of fragility fractures has been recognized as another significant diabetes complication [1].

According to a Rotterdam study, individuals with T2DM have a 69% increased risk of fractures when compared with healthy controls. Paradoxically, T2DM subjects had normal or even greater bone mineral density (BMD) [2]. This indicates a weakening of bone biomechanical competence beyond what can be measured by BMD. This disruption of bone may be brought about by alterations in bone turnover rate and collagen synthesis [3].

Testing of serum levels of bone turnover markers (BTMs), a noninvasive method of evaluating bone turnover status, is recognized as a promising tool in the evaluation of bone metabolism and quality by the National Osteoporosis Foundation. BTMs are classified as bone formation markers and as bone resorption markers [4].

Recent evidence suggests an intimate relationship between glycemic control and bone homeostasis. This includes accumulation of advanced glycation end products (AGEs), peroxisome proliferator-activated receptor gamma (PPARγ), the incretin hormones like glucose-dependent insulinotropic peptide (GIP), glucagon-like peptide 1 and 2 (GLP-1 and GLP-2), increased oxidative stress, microangiopathy and the bone-derived hormone osteocalcin and sclerostin [1].

A novel coordinator of bone and glucose homeostasis is osteoglycin. Osteoglycin is a basic component of vascular extracellular matrix which is expressed by cardiomyocytes, cardiac fibroblast, and vascular smooth muscle cells [5]. It participates mainly as a regulator of bone metabolism [6], as it is a bone-associated glycoprotein, expressed by osteoblasts. Metabolically, it may increase insulin secretion in the pancreas and decrease insulin resistance in muscle and liver [7].

Osteoglycin was shown to be expressed in myoblast, and this expression is increased by active 1,25 Vitamin D [8]. The influence of osteoglycin on bone turnover is conflicting as some studies show that it inhibits osteoblast differentiation and decreases bone mass, while others suggest that osteoglycin may increase osteoblast activity and maturation [6, 7].

We aimed to evaluate serum levels of bone turnover markers (BTMs) (markers of bone formation and bone resorption) and BMD at three sites (lumber, neck femur, and total femur) in middle-aged men with type 2 diabetes and to analyze the relationship between them. Also to evaluate serum osteoglycin as a novel marker and its relation to BTMs, BMD, and diabetic status.

## Materials and methods

This cross-sectional descriptive-analytic study was performed in the outpatient clinic at Kasr El Aini Center for Endocrinology and Diabetes. Ninety-one subjects were enrolled from January 2022 till September 2022, of whom seventy-eight were middle-aged male patients with T2DM attending the outpatient clinic and thirteen were nondiabetic, male volunteers as a control group. The observed power of the study is 1 which means that Type II error (β) is very low meaning that the probability of having a false negative result is about 0%. Also, the estimate of the effect size (Partial Eta Squared) and significance was very large (0.6). The Kasr Alainy School of Medicine Ethics Committee of Cairo University in Egypt revised and approved the study protocol. The number of approvals was MD-224-2021.

We recruited male type 2 diabetic patients between the ages of 40–65 years. Patients with chronic kidney disease, chronic liver disease, cancer, rheumatic disease, Cushing’s disease, and thyrotoxicosis were excluded. Participants taking medications that may influence bone metabolism were also excluded such as glucocorticoids, calcium, Vitamin D, and antiosteoporosis drugs.

The chosen patients were subjected to full history taking including (age, duration of diabetes, History of hypertension or current usage of antihypertensive drugs, History of ischemic heart disease (IHD), Special smoking history) and complete physical examination. They were also subjected to:

1. Height and weight from which body mass index (BMI) was calculated as the weight divided by the height squared (Kg/m²).
2. Waist circumference was measured with a measuring tape at a point midway between the costal margin and iliac crest in the midaxillary line with the subject standing and breathing normal [9].

Laboratory tests were performed to measure their glycated hemoglobin (HbA1c), Homeostasis Model Assessment of Insulin Resistance (HOMA IR), BTMs (serum procollagen type 1 N-terminal propeptide [P1NP] and serum C-terminal telopeptides of type I collagen [CTX]), serum osteoglycin level, serum parathyroid hormone (PTH) and 25 hydroxy vitamin D (Vit D).

HOMA IR Test: The level of insulin resistance was determined in serum using an insulin resistance ELISA Kit that was provided by Glory Science Co., Ltd (San Diego, USA). CATALOG No: X1307. The level of P1NP was determined in serum using the P1NP ELISA kit (Cat: ELK5402), provided by ELK Biotechnology (USA). The sensitivity of the kit was 0.91 ng/ml. The level of CTX was detected in serum using an ELISA kit provided by ELK Biotechnology, Cat: ELK8623 (USA). The Detection range was 0.63-40 ng/ml. The level of osteoglycin was determined in serum using an ELISA kit that was supplied by ELK Biotechnology (USA), Cat: ELK3335. The test principle applied in this kit is Sandwich enzyme immunoassay. The level of PTH was determined in serum using a PTH ELISA kit (Cat: ELK2427), provided by ELK Biotechnology (USA). The sensitivity of the kit was 4.95 pg/mL. The level of vitamin D was determined in serum using Total 25-OH Vitamin D EIA Kit Enzyme Immunoassay (EIA) that was provided by Epitope Diagnostic Inc. (San Diego, USA). Cat. No KT 715.

DEXA scan for assessment of BMD was done at three different sites: lumbar spine, neck femur, and total femur. DEXA produces a so-called R-value, which is the ratio between the attenuation coefficients at the two energy levels. R-value is constant for bone and fat in all individuals, although it varies for soft tissue as it depends on the patient’s composition. If a subject has a high fat percentage, their R-value will be lower than that of a subject with a high lean mass percentage. Using the DEXA method allows for distinguishing between three different compartments based on their X-ray attenuation properties: bone mineral content (BMC); lipids (triglycerides, phospholipid membranes, organ, marrow, and subcutaneous adipose), which is the so-called fat mass (FM); and lipid-free soft tissue, which is the lean mass (LM) [10].

### Statistical analysis

The statistical software for the social sciences (SPSS) version 28 (IBM Corp., Armonk, NY, USA) was used to code and enter the data. For quantitative variables, mean and standard deviation were used to summarize the data, and for categorical variables, frequencies (the number of cases) and relative frequencies (percentages) were used. Unpaired t-tests were used to compare two groups, while analysis of variance (ANOVA) with multiple comparisons post hoc tests were used to compare more than two groups. The paired t-test was used to compare each group’s before and after data. An analysis using the Chi-square [2] test was done to compare categorical data. When the anticipated frequency is less than 5, an exact test was utilized instead. Correlation analysis is used to check whether there are significant correlations among continuous variables. The strength and direction of relationships are given by Pearson r. Correlation analysis, however, does not show which variables are independent and which are dependent variables. It does not show which variables affect the other. Standard Multiple Linear Regression Analysis was done to identify how much all independent variables affect the dependent variable as a group and individually. Statistics were considered significant for P-values under 0.05.

## Results

Table 1 provides a summary of the demographic and baseline laboratory information of the studied patients. In a trial to know the PTH response to vitamin D deficiency, we divided each group of vitamin D according to PTH level whether normal (14–65 pg/mL), low (< 14 pg/mL), or high (> 65 pg/mL). As shown in Table 2, 37.5% of patients with insufficient vitamin D levels had normal PTH levels and 67.24% of patients with vitamin D deficiency had normal PTH levels, while only 31.0% of them had a high PTH level. Based on the results of the DEXA scan patients were divided into three groups (osteoporosis, osteopenia, and normal). Results of the DEXA scan for the three different sites examined are shown in Table 3; only a few patients had osteopenia and even fewer patients had osteoporosis while the majority of patients had a normal scan.

**Table 1:** Demographic and baseline laboratory data of the studied patients

Variables	Diabetic Group (*n* = 78)	Control Group (*n* = 13)	*P*-Value
**Age (**Years**)** (Mean ± SD)	55.78 ± 7.36	48.8 ± 5.48	0.0016
**Duration of Diabetes (**Years**)** (Mean ± SD)	10.77 ± 9.02	0	-
**BMI** (kg/m²) (Mean ± SD)	30.24 ± 5.05	26.7 ± 3.7	0.0177
**Waist circumference** (cm) (Mean ± SD)	105.35 ± 14.98	96.3 ± 12.8	0.0429
**Smokers** (Number (%))	40 (51.3)	11 (84.6)	0.0259
**Hypertension** (Number (%))	20 (25.6)	3 (23.1)	0.8485
**Heart Disease** (Number (%))	12 (15.4)	0 (0)	-
**Oral Treatment only** (Number (%))	38 (48.7)	0 (0)	-
**Insulin Treatment only** (Number (%))	15 (19.2)	0 (0)	-
**Oral and Insulin Treatment** (Number (%))	24 (30.8)	0 (0)	-
**HbA1c** (Mean ± SD)	8.4 ± 1.6	5.4 ± 0.2	0.000
**HOMA-IR (U/ml)** (Mean ± SD)	2.8 ± 3.8	1.5 ± 0.99	0.214
**Vitamin D (ng/ml)** (Mean ± SD)	15.3 ± 8.3	28.5 ± 20.02	0.037
**PTH (pg/ml)** (Mean ± SD)	56.7 ± 24.6	47.5 ± 15.55	0.196
**CTX (ng/ml)** (Mean ± SD)	5.3 ± 5.2	2.8 ± 1.01	0.000
**P1NP (ng/ml)** (Mean ± SD)	157.2 ± 62.4	101.05 ± 70.4	0.004
**Osteoglycin (pg/ml)** (Mean ± SD)	13.5 ± 6.9	6.06 ± 3.7	0.000
**DEXA Lumbar Spine (SD)** (Mean ± SD)	-0.38 ± 1.3	-0.98 ± 0.95	0.109
**DEXA neck femur (SD)** (Mean ± SD)	-0.32 ± 1.4	-0.14 ± 1.4	0.67
**DEXA total femur (SD)** (Mean ± SD)	0.00 ± 1.3	-0.13 ± 1.5	0.743

BMI = Body mass index; HbA1c = glycated hemoglobin; HOMA IR = Homeostasis Model Assessment of Insulin Resistance; PTH = serum parathyroid hormone; CTX = serum C-terminal telopeptides of type I collagen; P1NP = serum procollagen type 1 N-terminal propeptide; SD = Standard Deviation

**Table 2:** Serum parathyroid hormone levels among different groups of 25 hydroxyvitamin D

Vit D groups		PTH groups			Total number of patients
		Low (< 14 pg/mL)	Normal (14–65 pg/mL)	High (> 65 pg/mL)	
**Normal** ≥ 30 ng/mL	Number of patients	0	4	0	4
	Percentage	0	100	0	
**Insufficient** ≥ 20 to < 30 ng/mL	Number of patients	1	6	9	16
	Percentage	6.25	37.5	56.25	
**Deficient** < 20 ng/mL	Number of patients	1	34	18	53
	Percentage	1.724	67.24	31.034	

PTH = serum parathyroid hormone; Vit D = 25 hydroxyvitamin D

**Table 3:** Analysis of the results OF DEXA scan

DEXA results	Diabetic Group (*n* = 78)			Control Group (*n* = 13)		
	Lumbar Spine (Number (%))	Neck Femur (Number (%))	Total Femur (Number (%))	Lumbar Spine (Number (%))	Neck Femur (Number (%))	Total Femur (Number (%))
Normal ≥ -1	50 (64.1)	53 (67.9)	63 (80.8)	6 (46.2)	8 (61.5)	10 (76.9)
Osteopenia -1 to -2.5	24 (30.8)	22 (28.2)	15 (19.2)	6 (46.2)	5 (38.5)	3 (23.2)
Osteoporosis ≤ -2.5	4 (5.1)	3 (3.8)	0 (0)	1 (7.7)	0 (0)	0 (0)

**Fig. 1:**
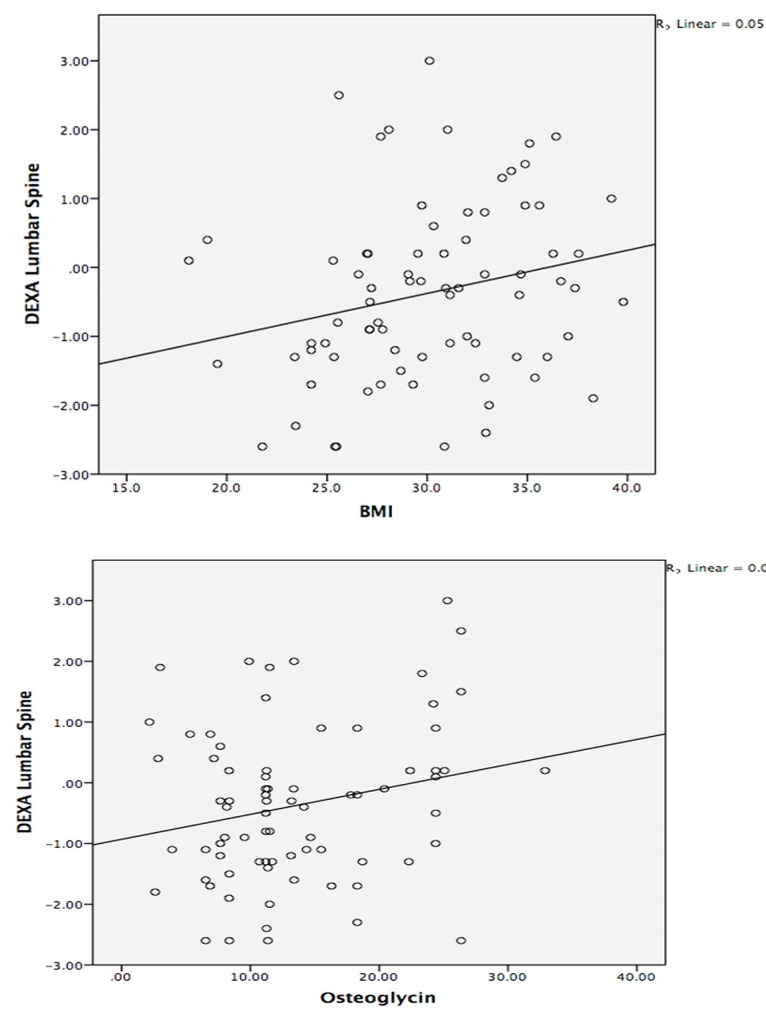
Correlation between body mass index, serum Osteoglycin and DEXA scan lumbar spine. BMI = Body mass index

**Fig. 2:**
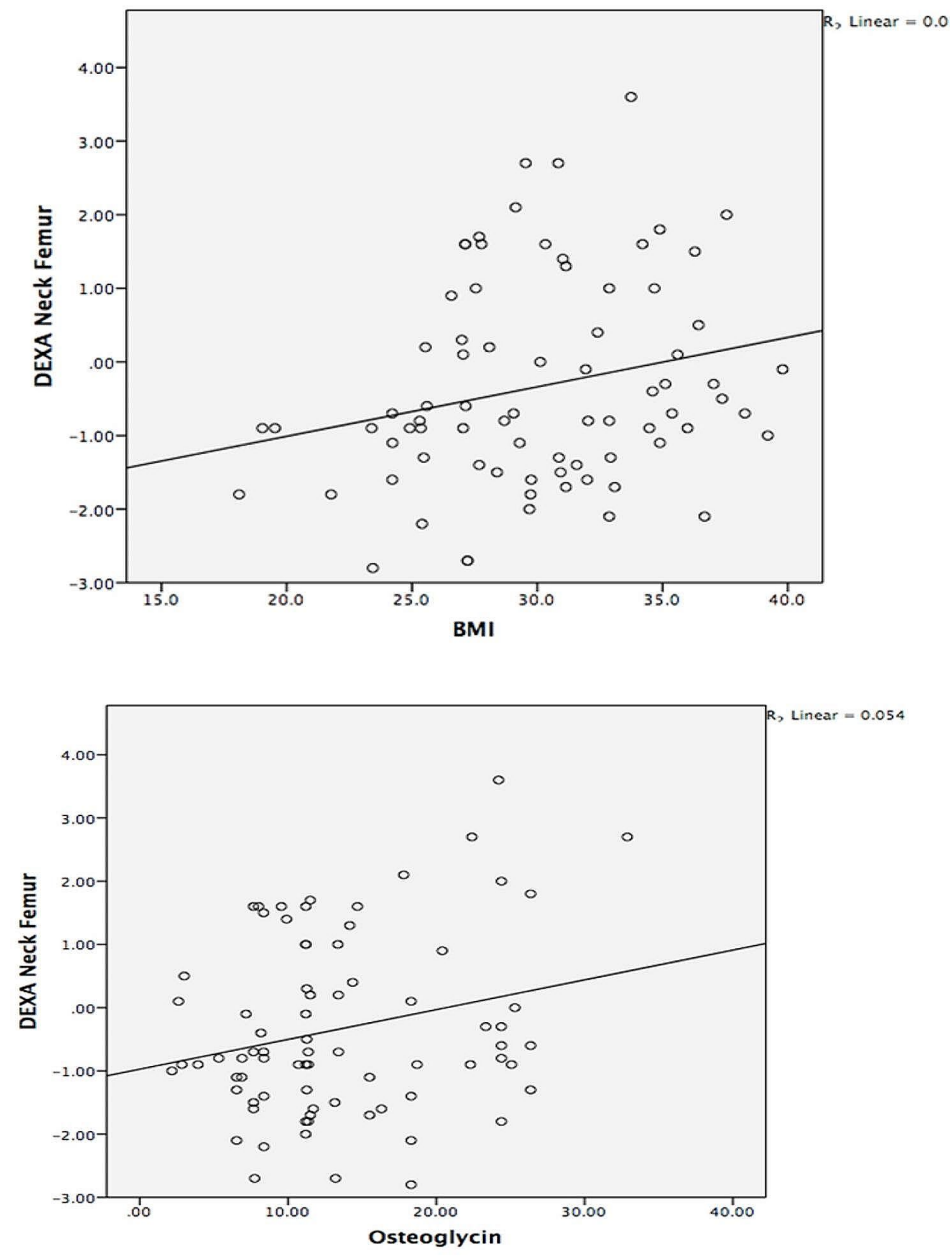
Correlation between body mass index, serum Osteoglycin, and DEXA scan neck femur. BMI = Body mass index

Correlation Analysis was conducted in Table 4 by using the Pearson correlation coefficient. BMI was positively correlated with waist circumference (*r* = 0.724; *P* = 0.000; < 0.001). HOMA-IR was negatively correlated with Vitamin D (*r* = -0.313; *P* = 0.006; < 0.001). CTX was negatively correlated with BMI (*r* = -0.233; *P* = 0.042; < 0.05) and waist circumference (*r* = -0.247; *P* = 0.030; < 0.05). P1NP was positively correlated with BMI (*r* = 0.225; *P* = 0.050; < 0.05) and negatively correlated with HOMA-IR (*r* = -0.225; *P* = 0.049; < 0.05). DEXA Lumbar Spine was positively correlated with BMI (*r* = 0.234; *P* = 0.042; < 0.05) (Fig. 1) and with waist circumference (*r* = 0.300; *P* = 0.008; < 0.001). DEXA Neck Femur was positively correlated with BMI (*r* = 0.230; *P* = 0.044; < 0.05) (Fig. 2), waist circumference (*r* = 0.241; *P* = 0.035; < 0.05), and Vitamin D (*r* = 0.258; *P* = 0.023; < 0.05), DEXA Total Femur was positively correlated with BMI (*r* = 0.265; *P* = 0.020; < 0.05). Osteoglycin was positively correlated with DEXA Neck Femur (*r* = 0.233; *P* = 0.041; < 0.05) (Fig. 2).

**Table 4:** Correlations of BMI, Waist circumference, HOMA-IR, and DEXA scan Different Variables among the studied patients

Variables		BMI, Waist circumference and HOMA-IR		
		BMI (kg/m²) (*n* = 78)	Waist circumference (cm) (*n* = 78)	HOMA-IR (*n* = 78)
BMI	r	**-**	0.724^**^	0.030
	*P*-value	**-**	0.000	0.797
Waist circumference	r	0.724^**^	**-**	-0.210
	*P*-value	0.000	**-**	0.066
HbA1c	r	0.077	-0.094	0.121
	*P*-value	0.504	0.414	0.296
Vitamin D (ng/ml)	r	0.079	0.001	-0.313^**^
	*P*-value	0.497	0.996	0.006
PTH (pg/ml)	r	0.081	-0.027	0.094
	*P*-value	0.484	0.813	0.414
CTX (ng/ml)	r	-0.233^*^	-0.247^*^	0.082
	*P*-value	0.042	0.030	0.478
P1NP (ng/ml)	r	0.225^*^	0.190	-0.225^*^
	*P*-value	0.050	0.098	0.049
Osteoglycin (pg/ml)	r	0.029	-0.030	0.070
	*P*-value	0.802	0.792	0.547
**Variables**		**DEXA scan**		
		**Lumbar Spine (SD)** (***n*** = 78)	**Neck Femur (SD)** (***n*** = 78)	**Total Femur (SD)** (***n*** = 78)
BMI (kg/m²)	r	0.234^*^	0.230^*^	0.265^*^
	*P*-value	0.042	0.044	0.020
Waist circumference(cm)	r	0.300^**^	0.241^*^	0.210
	*P*-value	0.008	0.035	0.066
HbA1c	r	-0.169	0.017	0.059
	*P*-value	0.144	0.885	0.611
HOMA-IR (U/ml)	r	-0.188	-0.203	-0.140
	*P*-value	0.104	0.076	0.225
Vitamin D (ng/ml)	r	-0.064	0.258^*^	0.217
	*P*-value	0.581	0.023	0.059
PTH (pg/ml)	r	-0.109	0.027	0.061
	*P*-value	0.346	0.818	0.597
CTX (ng/ml)	r	-0.082	0.061	0.026
	*P*-value	0.480	0.599	0.821
P1NP (ng/ml)	r	0.149	0.111	0.182
	*P*-value	0.199	0.338	0.112
Osteoglycin (pg/ml)	r	0.222	0.233^*^	0.199
	*P*-value	0.054	0.041	0.082

BMI = Body mass index; HbA1c = glycated hemoglobin; HOMA IR = Homeostasis Model Assessment of Insulin Resistance; PTH = serum parathyroid hormone; CTX = serum C-terminal telopeptides of type I collagen; P1NP = serum procollagen type 1 N-terminal propeptide; SD = Standard Deviation

**. Correlation is significant at the 0.01 level (2-tailed)

*. Correlation is significant at the 0.05 level (2-tailed)

The multiple regression analysis for the Osteoglycin model showed that none of the independent variables had a significant influence on the level of Osteoglycin. The multiple regression analysis for the DEXA parameters model showed that only BMI and Osteoglycin have a significant effect on the DEXA lumbar spine. Based on this regression model, when the BMI increases by 1, DEXA lumbar spine increases by 0.071. When Osteoglycin increases by 1, DEXA lumbar spine increases by 0.050. Although only BMI and Osteoglycin influenced the other DEXA parameters, as seen in the previous regression models, they do not influence DEXA Total Femur.

## Discussion

Diabetes is a metabolic disease with complications that affect almost all body systems. However, the impact of diabetes on bone is frequently underestimated [11]. The relative risk of hip fracture in patients with T2DM has been estimated at 2.8 for men and 2.1 for women, both are statistically significant. These findings position hip fracture as an unrecognized chronic complication of T2DM [12].

Bone strength can be defined as a reflection of the integration of bone density and quality. Bone density is determined by peak bone mass and amount of bone loss. Bone quality describes aspects of bone composition and structure that contribute to bone strength independently of bone mineral density. These include bone turnover, microarchitecture, mineralization, microdamage, and the composition of bone matrix and mineral [13].

DEXA measured bone mineral density accounts for 60–70% of the variation in bone strength and bone quality accounts for about 20% [14]. Thus, to evaluate bone health in people with T2DM, bone strength, including BMD and bone quality (bone turnover), should be assessed [15].

Testing of serum levels of BTMs is a noninvasive method in evaluating bone turnover status. BTMs are classified as bone formation markers, and bone resorption markers [16]. BTMs are recognized as promising tools in the evaluation of bone metabolism and quality by the National Osteoporosis Foundation [17]. In clinical practice, the use of BMD examination was always restricted by a limited number of instruments and a relatively longer follow-up period, while BTM testing is rather more convenient. Moreover, the changes in serum levels of BTMs are usually faster than levels of BMD. Consequently, the analysis of the relationship between BTMs and BMD in patients with T2DM might contribute to the prediction of changes in BMD levels according to variations in serum levels of BTMs in clinical practice [15]. Some bone metabolic hormones also influence bone metabolism, such as parathyroid hormone (PTH) and 25‐hydroxyvitamin D (25[OH]D) [15].

To evaluate bone metabolism in patients with T2DM, BMD, and levels of bone turnover markers were included in our study. Further, we tried to find the relationship between them and to find out if they interacted with each other. Gender also appears to have an important effect on the relation between BMD and T2DM. Our study was performed on a group of male patients in the age range of 40–65, to avoid the interference of the effect of female sex steroids and aging on BMD [18, 19].

In our study, BMD levels were tested by DEXA at three different sites including, the lumbar spine, neck femur, and total femur. P1NP was tested as a marker of bone formation and CTX as a marker of bone resorption. P1NP and CTX have been suggested by the International Osteoporosis Foundation as the reference BTMs when exploring bone formation and resorption in clinical and research settings [17].

The results of our study showed that 62.8% of patients had normal DEXA scan in the lumbar spine, 67.9% had normal DEXA scan at the neck femur and 80.8% had normal DEXA scan in the total femur. These findings are like the control group where 61.5% had a normal DEXA scan at the neck femur and 76.9% had a normal DEXA scan at the total femur but only 46.2% had a normal DEXA scan at the lumbar spine. Studies on BMD investigated in T2DM showed contradictory results with higher, lower, or similar values in comparison with healthy control subjects [20–22].

Our study showed that BMI was positively correlated with BMD at both the lumbar spine and neck femur. Based on our regression model, when the BMI increases by 1, DEXA at each of these sites increases by 0.071. Two meta-analyses, by Ma and colleagues in 2012 and a previous one by Vestergaard in 2007, both showed that, like our study, BMI was positively correlated with BMD. The mechanisms of the association of BMI with BMD in vivo may include increased loading, decreased bone turnover, and several adipokines released from adipose tissue [19, 22].

The insulin resistance typical of T2DM occurs also in bone tissue, where insulin does not exert its full anabolic effect. In our study, no significant correlation was found between HOMA-IR and BMD at the three different sites. By applying multiple regression analysis no significant relationship was found between the two variables. In a previous study, an inverse relationship between bone strength and insulin resistance measured by HOMA-IR was reported in peri-menopausal women [23].

Vitamin D insufficiency and deficiency in T2DM have been reported in many studies. Vitamin D deficiency has been implicated in decreased insulin secretion and increased insulin resistance, and more recently with the development of T2DM [24]. As regards vitamin D levels, 74.4% of our patients had vitamin D deficiency and 20.5% had insufficient vitamin D levels. Our study showed a significant negative correlation between vitamin D and insulin resistance as assessed by HOMA-IR. Although vitamin D was low in our study, PTH was not high. While 74.4% of our patients had vitamin D deficiency, 67.2% of them had normal PTH levels and only 31% had a high PTH. This was also the case among the group of patients with insufficient vitamin D levels, where 37.5% of them had a normal PTH level. Vitamin D deficiency is believed to cause secondary hyperparathyroidism, leading to an increase in bone turnover and bone loss. A negative correlation exists between serum parathyroid hormone and serum 25-hydroxyvitamin D [25]. It has been suggested that a state of relative or subclinical hypoparathyroidism could contribute to low bone turnover in patients with T2DM [26]. In our study, no such relation was detected between vitamin D, PTH, and BTMs.

Bone turnover markers are generally reduced in patients with T2DM [27]. In a meta-analysis, a state of low bone turnover was determined in patients with diabetes as both markers of bone formation and bone resorption were decreased [28]. Low bone turnover was confirmed by Starup-Linde and colleagues in 2021 where they found lower levels of CTX and P1NP in people with T2DM compared to control [29]. It has been hypothesized that low bone turnover in diabetes compromises the healing of micro-fractures due to the suppression of bone formation and that the accumulation of micro-fractures may predispose individuals with diabetes to fractures [30].

Some studies revealed a lowering of only one marker, either bone resorption or formation. Our study showed contradictory results, where both markers of bone formation and bone resorption were increased indicating a state of increased bone turnover. It is stated that increased bone turnover increases the proportion of newly formed bone, which is less well-mineralized than mature bone and has fewer post-translational modifications of bone collagen, such as cross-linking and B isomerization [17].

In our study BTMs showed a correlation with BMI, where a significant positive correlation was evident between P1NP and BMI, and a significant negative correlation was found between CTX and BMI. Like our results lower levels of CTX and higher femoral BMD were demonstrated in overweight postmenopausal women with T2DM by Bilić-Ćurčić in 2017 [31]. Also, the results of Safarova in 2019 revealed a higher BMI in individuals with low serum CTX [32]. A high BMI is known to increase BMD by decreasing bone turnover [33].

Serum osteoglycin level in our study was significantly higher in T2DM as compared to the control group. It was also positively correlated to DEXA neck femur. Regression analysis confirmed this and in addition, showed a positive association of osteoglycin with DEXA lumbar spine. However, no correlation was found between osteoglycin and DEXA total femur.

Osteoglycin in our study showed no correlation with BTMs, HbA1c, insulin resistance, or any other variables. Similar to our results previous studies found no evidence of an association between osteoglycine and BTMs, glucose, or HbA1c [29, 34]. In contrast to our results, in one of these studies, osteoglycine levels were not associated with BMD at the hip, femur, lumber spine, or distal forearm [29], even more in the other study osteoglycine levels were associated with decreased BMD and the presence of vertebral fractures [34]. These findings may suggest osteoglycine to be a marker of low BMD and vertebral fractures in T2DM which goes against our results [34].

Results of our study do not fall under one category and so no solid conclusion can be drawn and generalized in the use of BTMs as markers for the detection of bone turnover in diabetic patients. The small sample size was one of our study’s key limitations. Our results confirmed that patients with T2DM have preserved BMD and that osteoglycine and BMI seem to be regulators of BMD. Abnormal bone turnover was also observed in our study where both markers of bone formation and bone resorption were increased indicating a state of increased bone turnover in T2DM. However, additional research and data collection are suggested before implementing them in clinical settings. They should be examined carefully and only in conjunction with clinical data.

## Conclusion

BMI and Serum osteoglycin have a significant positive effect on BMD. Both markers of bone formation and bone resorption were increased indicating a state of increased bone turnover in T2DM.

## Data Availability

No datasets were generated or analysed during the current study.
